# Using multistakeholder dialogues to assess policies, programmes and progress for women’s, children’s and adolescents’ health

**DOI:** 10.2471/BLT.16.171710

**Published:** 2016-05-02

**Authors:** Laura Frost, Rachael Hinton, Beth Anne Pratt, John Murray, Sharon Arscott-Mills, Susan Jack, Andres de Francisco, Shyama Kuruvilla

**Affiliations:** a Global Health Insights, New York, United States of America (USA).; b The Partnership for Maternal, Newborn & Child Health, avenue Appia 20, 1211 Geneva 27, Switzerland.; c Independent Consultant, Iowa City, USA.; d ICF International, Fairfax, USA.; e Centre for International Health, University of Otago, Dunedin, New Zealand.; f World Health Organization, Geneva, Switzerland.

Stakeholders from a range of sectors – including health, finance, planning, water and sanitation, nutrition and education – and from diverse constituencies – including government, nongovernmental organizations, private sector and academic institutions – all contribute to the improvement of women’s, children’s and adolescents’ health. Multistakeholder dialogues are structured processes used to bring stakeholders together to develop a shared understanding of issues, evidence and plans of action.^1^ The sustainable development goals (SDGs) and the *Global strategy for women’s, children’s and adolescents’ health (2016–2030) *emphasize the importance of multistakeholder and cross-sector collaboration.^2^^,^^3^ Multistakeholder dialogues can facilitate these processes, and their benefits and challenges have been shown in a variety of sectors and contexts.^1^ Few multistakeholder dialogues have been systematically documented and evaluated, which is required to understand how they can be most effectively undertaken to support implementation and evaluation.

This paper describes multistakeholder dialogues conducted as part of the *Success factors for women’s and children’s health* studies.^4^^,^^5^ When the studies started in 2012, only 10 low- and middle-income countries were on track to meet both millennium development goals (MDGs) 4 and 5A, to reduce the under-five mortality rate by two-thirds and reduce the maternal mortality ratio by three quarters, respectively. These countries were Bangladesh, Cambodia, China, Egypt, Ethiopia, the Lao People's Democratic Republic, Nepal, Peru, Rwanda and Viet Nam.^5^ Between 2014 and 2015, these countries conducted dialogues to understand which policies or programmes, both within and outside the health sector, contributed to progress. They then documented the results in country policy reports.^4^ The policy and programme findings across the country multistakeholder dialogues are published elsewhere.^6^

Here, we describe the processes, identify strengths and challenges and highlight key lessons for future efforts, by drawing on the perspectives of those who participated in the design and implementation of the dialogues.

In total, 407 stakeholders across the 10 countries engaged in the dialogues. While initiated by international study partners, the dialogues in each country were led by health ministries and convened by World Health Organization (WHO) country teams (or an organization identified by WHO) with key development partners.^4^ National consultants in most countries, three international consultants and international partner organizations provided technical support.

The dialogues followed three phases (Table 1). Phase 1 was preparatory. International partners contacted national conveners and provided a background literature and data review. In most countries, the convening organization contracted a national consultant to facilitate the dialogues and update the background information. A first draft report, on the policies and programmes potentially associated with mortality reductions, was distributed to participants.

**Table 1:** Overview of a multistakeholder dialogue process and time required for each step

Phase	Task	Requirement^a^
**Phase 1: Preparatory work**	– Facilitation by international and national coordinating agency, contacting key stakeholders and consultants to define objectives of the dialogue, alignment with national processes and events and meeting logistics (define dates, identify stakeholder participants, send invitations, etc.).	15 days
	– National and international consultants’ literature and data review, develop background document.	20 days
	– Collect additional data (e.g. stakeholder interviews) and share background document with participants for quick review.	20 days
	– Update background document.	10 days
**Phase 2: Convening and facilitation of the dialogues**	– Organize meeting agenda and logistics.	5 days
	– Meeting and reaching agreement on findings and follow-up action and communication ideas, linking with national, regional and global processes.	2 days
	– Travel for national and international participants and consultants to attend the dialogue. National travel as required: 25–30 participants, International travel: 2–3 people.	Travel costs for participants
**Phase 3: Dissemination and follow-up**	– Validate findings and prepare final country policy report.	20 days
	– Develop plan for dissemination, including relevant media and follow-up action linking to national, regional and global processes.	5 days
	– Communications-related contracts such as layout and design of reports for publication, drafting press releases.	25 days
	– Communication by national coordinating agency, including publication clearances, media outreach and links with stakeholders and related policy processes.	15 days
	– Communication by international coordinating agency, including publication clearances, media outreach and links with stakeholders and related policy processes.	15 days
	– Follow-up action and implementation, external evaluation and research.	NA^b^

NA: not available

^a^ Time required to complete the tasks and related costs will vary by country. For the Success Factors’ series of country multistakeholder dialogues the average cost per country was approximately 125 000 United States dollars. Additional resources would need to be allocated for participant and external evaluations, for implementation of follow up activities, and for further related research on the effects and impacts of the MSD process. Some costs may also be absorbed as staff and recurrent costs, particularly for the national and international agencies and government staff.

^b^ Data on time and related costs were not available for follow-up action and implementation activities; external evaluation and related implementation research are important considerations but were not included in this initiative and would require substantial additional costs.

Phase 2 was conducting the multistakeholder meetings.^4^ Participants agreed on four plausibility criteria to determine which policy and programme inputs were likely to be associated with mortality reductions (Box 1). In most countries the meetings were held over two days. In one country, individual interviews replaced the meeting because of contextual constraints. In two countries, follow-up meetings were convened to ensure participation from sectors outside health.

Box 1 **Plausibility criteria for multistakeholders to identify policy and programme inputs as success factors for reducing child and maternal mortality**

- Potential impact: likely to have contributed to mortality reduction based on an impact framework and available data.
- Temporal association: had been implemented long enough to have influenced mortality.
- Scale: had reached a large enough target population to influence mortality.
- Consensus: broad agreement between key stakeholders within and outside the health sector.

Phase 3 was dissemination of findings and follow up. Findings were further validated using key informant interviews in some countries. The final country policy reports were prepared by the international consultants, reviewed by national stakeholders, and signed-off by health ministries.^4^ Plans for dissemination and follow-up action varied in each country.

The phases, timing and costs associated with the multistakeholder dialogues are detailed in Table 1.

The seven lessons learnt are based on convener and consultant experiences, one-on-one interviews conducted by the lead author and participant feedback from country meetings and reports.^4^ Interview methods were approved by the WHO Ethics Review Committee (RPC695) and interviewees gave consent to participate. The lessons are assessed against best practices set out in the Multistakeholder Dialogue Guide for Women’s and Children’s Health.^1^ Data were analysed using qualitative content analysis in which codes are assigned based on a review of the text and then used to derive thematic categories.^7^

The first lesson is the need for systematic analysis and time to identify participants and conduct key stakeholder interviews before the meetings.^1^ We initially had underestimated the time and coordination required for this preparatory work. Individual interviews have multiple benefits including: identifying additional sources of data; in depth exploration of specific policies, programmes and actors’ roles; and improving buy-in to the dialogue, particularly from non-health sector stakeholders. One interviewee pointed to a main challenge being the significant commitment required. “We invested substantial time in organizing and conducting the one-on-one meetings. The international and national consultants mostly contributed to that. Several staff from [the WHO country team] contributed as interviewers of high-level government persons… and facilitated the appointments. It was a good collective effort.”

The second lesson is the importance and the challenge of engaging other sectors in the dialogue. As one national convener stated: “It was very difficult to get the non-health sector involvement. You have to use your connections, personal relationships. I would recommend getting the highest level in the health ministries to issue the invitations to the other sectors.” Including items from other sectors on the agenda helped engagement. In Peru, the meeting included a child nutrition review with participants from health and other ministries. The dialogues provided a relatively rare opportunity for cross-sectoral discussions on health-related topics. One participant said: “We were able to reinforce the point that the social determinants of these [health and development] problems are really similar and that it is important to avoid divisions.”

The third lesson is the need to give sufficient attention to the background evidence review, data sources and triangulation. Identifying data sources required significant time and effort, however, a framework outlining data requirements helped organize and streamline data collection. We expected that global data comparable across countries would provide a satisfactory background for the dialogues when in fact, considerable time had to be spent reaching consensus on appropriate sources of mortality and coverage data on a global, national or subnational level. When there were discrepancies, participants mostly preferred to use country-based data – even if these showed less progress – as they felt this information was more reliable and relevant to follow-up actions. Further, local data on policy, planning and systems inputs were often available, but had not been systematically reviewed during phase 1. In Bangladesh, data from national longitudinal databases and operational research helped quantify links between health outcomes and particular policy inputs.

The fourth lesson is the importance of developing and using agreed plausibility criteria for the dialogues (Box 1). The criteria helped to address the challenge of defining best practices of the policy and programme management that contributed to mortality reductions. For instance, participants in Nepal used the temporal association criterion to identify different inputs and stages in long-term maternal mortality declines. The safe abortion policy in the early 2000s was important, but maternal mortality declines had started in the early 1990s due to other factors including reduced fertility rates, spousal separation (due to migrant work) and better antenatal care. In the Lao People's Democratic Republic, the scale criterion was important. Although coverage of health interventions had remained relatively low over time, participants determined that factors such as female literacy, infrastructure development and poverty reduction were critical to maternal and child mortality reductions.

The fifth lesson is the challenge of early planning and resource allocation for follow-up activities and implementation research. The country policy reports from the dialogues were presented at various national health meetings and have been used by policy-makers and media as reference documents. However, interviewees reported less use of the findings in policies and programmes than hoped for due to limited dissemination, budget and planning for follow-up actions. External evaluations of the dialogues and related implementation research are important considerations. However, these evaluations were not included in this initiative and would require substantial additional costs that need to be factored in the early planning phase.

The sixth lesson is key stakeholders are more likely to value and engage with the dialogues when they are integrated with ongoing national planning and policy cycles and linked to related regional and global processes to improve women’s, children’s and adolescents’ health. A main barrier to achieving these linkages in some countries was the limited timeframe to undertake the dialogue process. Linking dialogues to ongoing policy processes also requires considerable commitment, but is important for strengthening stakeholder engagement and ownership. Those countries in which the health ministry and WHO country team showed sustained engagement throughout the dialogue experienced wider involvement from key stakeholders and more commitment to long-term outcomes. While initially sceptical, many participants saw the dialogues as a unique opportunity to discuss national progress and tell their own story to an international audience. Indicating this sense of ownership, the health ministries in each country were lead authors of the country policy reports.^4^ Ministerial representatives presented the findings at the Partnership for Maternal, Newborn & Child Health’s Partners’ Forum in South Africa in 2014^8^ and provided media interviews that brought attention to the importance of country leadership and strategies used to accelerate progress.^9^ The Success Factors’ studies also informed the development of the global strategy.^2^

The seventh lesson is that leadership at all levels is essential to advance multistakeholder dialogues.^1^ We did not fully anticipate the significant commitment required to build a coordinated effort for the dialogues. Leadership and commitment from the health ministries, country partners and national consultants, global and regional partner support, and strong engagement of the international consultants ensured that all phases were conducted within a limited timeframe. Consultants and conveners with deep technical knowledge of the topics, local system and familiarity with the stakeholders were critical to moving forward the dialogues, for example in China, Egypt and the Lao People's Democratic Republic. As one convener explained, “All stakeholders were actively participating in the meeting, discussing and giving their views willingly and positively, especially since the national consultant was known and respected by most of them.” In other countries – such as Ethiopia and Rwanda – the health ministries played a pivotal role.

This paper outlined the process and lessons learnt from multistakeholder dialogues that examined the evidence and identified policies and programmes that contributed to reducing maternal and child mortality during the MDG era. These dialogues can support multistakeholder analysis, action and accountability towards implementing the global strategy, improving women’s, children’s, and adolescents’ health and achieving the SDGs.
